# Is Primary Care Patient Experience Associated with Provider-Patient Language Concordance and Use of Interpreters for Spanish-preferring Patients: A Systematic Literature Review

**DOI:** 10.1007/s40615-024-01951-z

**Published:** 2024-03-05

**Authors:** Denise D. Quigley, Nabeel Qureshi, Zachary Predmore, Yareliz Diaz, Ron D. Hays

**Affiliations:** 1 RAND Corporation, 1776 Main Street, Santa Monica, CA 90407-2138, USA; 2 RAND Corporation, 20 Park Plaza, Suite 910, Boston, MA, USA; 3 David Geffen School of Medicine & Department of Medicine, UCLA, 1100 Glendon Avenue, Los Angeles, CA 90024-1736, USA

**Keywords:** Patient experience, Clinician and group CAHPS survey, Primary care, Quality of care

## Abstract

**Background:**

Healthcare provided by a bilingual provider or with the assistance of an interpreter improves care quality; however, their associations with patient experience are unknown. We reviewed associations of patient experience with provider-patient language concordance (LC) and use of interpreters for Spanish-preferring patients.

**Method:**

We reviewed articles from academic databases 2005–2023 following the Preferred Reporting Items for Systematic Reviews and Meta-Analyses guidelines and Joanna Briggs Institute Checklists to rate study quality. We reviewed 217 (of 2193) articles, yielding 17 for inclusion.

**Results:**

Of the 17 included articles, most articles focused on primary (*n* = 6 studies) or pediatric care (*n* = 5). All were cross-sectional, collecting data by self-administered surveys (*n* = 7) or interviews (*n* = 4). Most assessed the relationship between LC or interpreter use and patient experience by cross-sectional associations (*n* = 13). Two compared subgroups, and two provided descriptive insights into the conversational content (provider-interpreter-patient). None evaluated interventions, so evidence on effective strategies is lacking. LC for Spanish-preferring patients was a mix of null findings (*n* = 4) and associations with better patient experience (*n* = 3) (e.g., receiving diet/exercise counseling and better provider communication). Evidence on interpreter use indicated better (*n* = 2), worse (*n* = 2), and no association (*n* = 2) with patient experience. Associations between Spanish-language preference and patient experience were not significant (*n* = 5) or indicated worse experience (*n* = 4) (e.g., long waits, problems getting appointments, and not understanding nurses).

**Conclusion:**

LC is associated with better patient experience. Using interpreters is associated with better patient experience but only with high-quality interpreters. Strategies are needed to eliminate disparities and enhance communication for all Spanish-preferring primary care patients, whether with a bilingual provider or an interpreter.

## Introduction

As of 2020, 19% (62.1 million) of the United States (U.S.) population was Hispanic, increasing from 16% (50.5 million) in 2010 [1]. The Hispanic population is estimated to reach 106 million, consisting of roughly 30% of the U.S. population, by 2050 [2]. In addition, the 2020 U.S. census estimated that 28% of Hispanics in the U.S. have limited English proficiency (29.7 million). These demographic shifts have consequences for high-quality healthcare delivery. As the Hispanic population grows, with a significant portion preferring Spanish due to limited English proficiency, healthcare organizations will continue to be faced with the challenges of ensuring high-quality patient-provider communication and determining whether they are providing high-quality language services to their patients and families.

Hospitals and other healthcare organizations that receive federal funding, such as Medicare or Medicaid payments, are required to provide formal professional interpreter services to individuals with limited English proficiency through Title VI of the Civil Rights Act of 1964 [3] and Executive Order 13,166 [4].

Despite this Federal requirement, non-English-preferring patients are not always ensured access and support from interpreters when needed. Nationally, fewer than one-third of outpatient physicians reported regularly using trained professional interpreters when communicating with non-English preferring patients [5], based on the 2016 national cultural and linguistically appropriate service (CLAS) physician survey prepared by the National Committee for Quality Assurance, which is a cross-sectional survey of non-federally employed, office-based physicians conducted by the Centers for Disease Control and Prevention [6]. This is to some extent due to the variety of strategies employed for supporting communication for non-English-preferring patients: use of bilingual providers, formal professional interpreters (in person, via phone, via video), ad hoc medical interpreters (i.e., bilingual employees such as nurses, clerks), or interpretation through family or friends.

Poor-quality communication with physicians and nurses due to limited English proficiency of patients leads to worse patient experiences with care and negative clinical experiences [7–10]. Language barriers, inadequate interpreter use, and poor provider-patient communication or miscommunication are associated with an increased risk of serious medical events such as prescription drug complications [11]. This can occur for many reasons, including miscommunications between providers and patients about symptoms that lead to misdiagnosis or miscommunications after a correct diagnosis that result in inadequate or inaccurate patient education about medications [12, 13]. Effective communication between providers and patients is a critical aspect of delivering high-quality care [14–21].

Many strategies are suboptimal in the primary care setting. For example, providing on-site professional interpreters in busy clinics may be expensive and lead to delays in care. Using family and friends as interpreters can compromise patient confidentiality and may result in misinterpretation of medical terms. Using bilingual employees at the clinic takes these staff away from their assigned work, impacting the flow and pace of providing care. There is evidence that a language-concordant physician is preferable to an interpreter for receiving the best medical care and care experience [22–24], but the pool of potential providers who are qualified as bilingual is often not adequate [25].

### Previous Reviews on Use of Interpreters

The most current literature reviews, both conducted more than 15 years ago, provided evidence on use of interpreters in primary care in the U.S. A literature review from 2005 on the impact of interpreter services in the U.S. included 36 articles (11 studies on primary care) [26–34] published between 1996 and 2003 and found that the overall quality of care is improved by use of either formal interpreter services or care from a bilingual provider [35]. One included study found that patients with limited English proficiency who needed but did not receive formal interpreter services did not have a good understanding of their diagnosis or treatment plan [26]. Furthermore, three of the studies indicated that when similar patients received ad hoc interpreter services, those interpreters often did not relay all the information shared by the provider, potentially leading to negative clinical consequences [27, 28, 36]. Two studies found that the use of interpreters was associated with less time for the patient to voice their concerns because translation time had the provider spend more time gathering the same information they gather with English-preferring patients [31, 36]. Overall, this review found evidence that care by a bilingual provider and care with the support of a formal interpreter were the two most promising strategies but acknowledged that future research is needed comparing whether different interpreter types have differential effects on patient experiences of care.

The second literature review from 2007 compared the use of professional interpreters to ad hoc interpreters for limited English proficient patients in the U.S. This review included 28 articles (8 on primary care) [28–32, 37–39] published between 1996 and 2005 and found that professional interpreters improved the quality of care by reducing communication errors, increasing patient understanding of their care, and improved clinical outcomes and overall patient experiences of care [40]. These patterns were similar, but weaker for the use of mixed interpreter groups (ad hoc and professional), suggesting that there is less benefit from ad hoc interpreters compared to professional interpreters. In addition, Kuo and Fagan (1999) reported that both providers and patients were more satisfied with professional in-person interpreters than with ad hoc staff interpreters. The evidence in this review showed strong benefit for the use of professional interpreters; however, it was noted that the quality of interpretation varies by the competency of the interpreter.

### Previous Reviews on Language Concordance

The most current literature review on patient-provider language concordance from 2019 included eight [41–48] studies on primary care in the U.S. [43]. One study found that language concordance for Spanish-preferring patients was not associated with any differences in perceived quality of well child [41] visits. Additionally, two studies [42, 44] found that language concordance is associated with more Spanish-preferring patients discussing lifestyle modifications with their providers and having more accurate recall of changes discussed at healthcare visits. Yet, another study found language concordance to be associated with a decrease in language barriers for patients wanting to communicate with their provider after hours [48]. The limited evidence on language concordance indicates a need to review evidence on the impact of language concordance on patient experience for Spanish-preferring patients in primary care settings.

Research to supply healthcare organizations with effective language support strategies for primary care is still needed and increasingly in demand across the U.S. To update the review of evidence, we examined and synthesized articles published since these previous reviews on the use of medical interpreter services and language concordance during primary care visits with Spanish-preferring patients on patient experience. The aim of the review is also to examine the associations of patient experience with provider-patient language concordance (LC) and use of interpreters for Spanish-preferring patients in primary care as well as to identify strategies that have been shown to improve the experience of Spanish-preferring patients in primary care.

## Methods

We adhered to the Preferred Reporting Items for Systematic Reviews and Meta-Analyses (PRISMA) guidelines (see Checklist Table, Online Resource 1, which is PRISMA checklist) [49, 50].

### Data Sources and Searches

We applied a structured search strategy to PubMed (using OVID Medline), Web of Science, Scopus, Cochrane Database of Systematic Reviews (via Wiley), APA PsycInfo, Cumulative Index of Nursing and Allied Health Literature (CINAHL), and a search of the Cochrane Library to identify peer-reviewed U.S. English-language articles from January 1, 2005, to January 31, 2023. Online Resource 2 (see Online Resource 2, which shows the search strategy) provides our keywords and Boolean operator strategy. To be included, an article needed at least one primary care term, one language/interpreter term, and one patient experience term.

### Screening

We (blinded; Authors1,2,3) reviewed titles and abstracts of identified articles. After an initial 30-study review by the full team to establish consistency across reviewers, individual reviewers independently screened abstracts for inclusion. All titles and abstracts were double-reviewed (blinded; Authors1&2 or Authors1&3). If initial assessments differed, reviewers discussed discrepancies and resolved disagreements, including discussion of the rationale for each initial assessment, definitions of relevant criteria, and any needed updates to definitions/criteria used, in order to reach consensus on inclusion.

### Data Abstraction and Quality Assessment

Reviewers (blinded; Authors1,2,3) abstracted information into a form noting: first author and year; objective; and relevant findings; study design (descriptive, comparative, correlational, experimental); study type (randomized control, case control, cohort, cross-sectional); statistical approach; methods; control variables; sample size; type of ambulatory care; sample description; population description; main and secondary outcomes; patient experience measures and timeframe; languages evaluated; disparities evaluated, if any; and limitations.

**Fig. 1:**
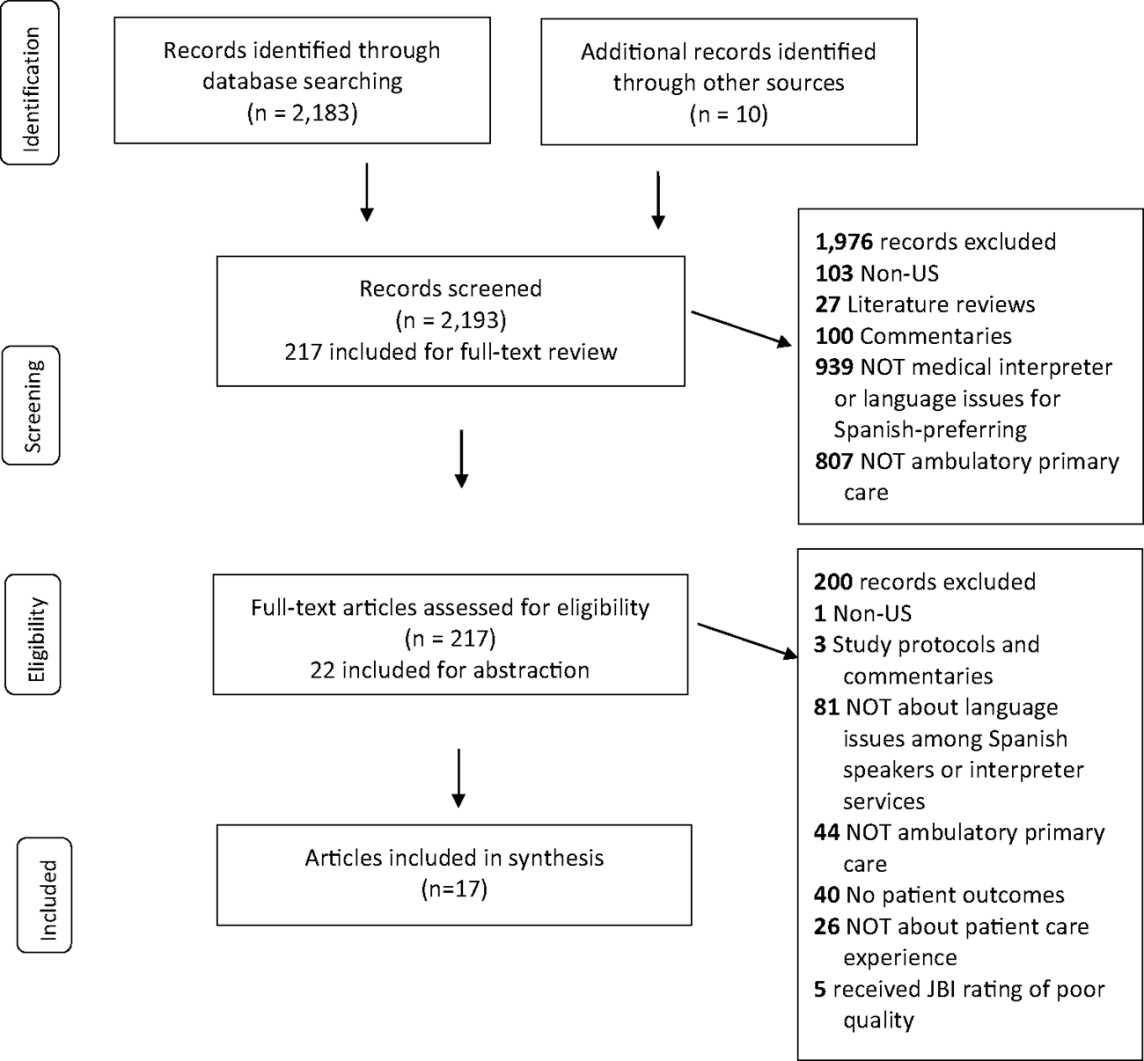
PRISMA flow diagram

Each of the three reviewers abstracted 10 articles after which the team met and discussed these articles and the abstraction process including a review of the definition of each abstracted data element, level of detail to capture/document, key important information for each abstracted data element, and how to flag articles that need discussion or questions concerning the inclusion of the article. Once we ensured that all three reviewers employed a similar abstraction approach, articles were assigned equally to individual reviewers for abstraction. After initial abstraction, a second reviewer examined each article to ensure the accuracy of abstracted content and, where necessary, discussed it with the team to reach consensus. Figure 1 details our screening and inclusion process.

The lead reviewer (blinded; Author1) provided a final review of the abstracted information to gain consistent detail (sample size, *p*-values) for constructing tables.

All twenty-two abstracted articles were cross-sectional, so we assessed their study quality using the Joanna Briggs Institute (JBI) Checklist for Analytical Cross-Sectional Studies for quantitative studies [51, 52] and the JBI Checklist for Qualitative Research for the two studies that used primarily qualitative research techniques [52]. We chose the JBI checklists because they are widely used and had checklists for many types of studies [53]. We excluded five cross-sectional studies because they did not possess at least six of eight cross-sectional study JBI Checklist for Analytical Cross-Sectional Studies elements. These excluded studies conducted and reported information about exposure and outcomes but did not report information on sample selection and/or did not control for confounding factors in analysis. We did not exclude any qualitative research studies because both met the threshold of at least seven of ten qualitative study JBI Checklist for Qualitative Research elements. Online Resource 3 and Online Resource 4 list the elements of the JBI Checklist for Analytical Cross-Sectional Studies and the JBI Checklist for Qualitative Research for each included article, respectively (see Online Resource 3 and Online Resource 4, which provides study quality data for included cross-sectional and qualitative studies, respectively).

## Results

As shown in Fig. 1, the search identified 2183 unique articles and a grey literature search identified 10 additional articles. After article and title screening and full-text review, 22 articles were abstracted and rated for study quality. During the screening and review process, we excluded articles that were not about language, medical interpreters, or Hispanic or Spanish-preferring patients (*n* = 1020), were not primary care (*n* = 851), did not include patient experience outcomes (*n* = 40), were not patient experience (*n* = 26), were not conducted in the U.S. (*n* = 104), were a literature review (*n* = 27), and were not empirical studies (i.e., commentaries) (*n* = 103). We excluded studies with poor study quality ratings (*n* = 5). In total, 17 articles remained for our synthesis.

### Examination of Included Studies

First, we review the types of primary care settings and populations, study types and design, and statistical methods used.

#### Type of Care and Patients

Eleven of 17 studies focused on general primary care settings including adult primary care (six studies) [44, 54–58] and pediatric primary care (five studies) [41, 59–62]. The other six studies examined a specific primary care patient population including primary care for patients with diabetes (four studies) [63–66], primary care focused on mental and behavioral health (one study) [67], and primary care interfacing with specialty care for adult patients who underwent teleretinal imaging within a federally qualified health center (FQHC) and were referred by their PCP to an ophthalmologist (one study) [68]. Online Resource 5 summarizes the main study topic, methods, population, measures, and data collection timeframe for each included study organized by setting (see Online Resource 5, which provides detailed study descriptions).

#### Study Types and Statistical Methods

All 17 included studies were cross-sectional, with seven studies using patient surveys [41, 55, 57, 58, 60–62] (two of which used the Clinician and Group Consumer Assessment of Healthcare Providers and Systems (CG-CAHPS) 2.0 patient experience survey [55, 62], including one study that also audio-recorded patient visits) [57], four patient phone interviews [63, 64, 67], two household surveys [56, 59], one conducted patient interviews post-visit [54], one conducted focus groups [66], and two used retrospective chart review [44, 68]. Fifteen included regression analysis (nine using linear regression [41, 55, 59–65], five logistic regression [44, 56, 58, 67, 68], and one negative binomial regression) [57]. Of the 15 studies for which investigators controlled for variables in the modeling, 15 [41, 44, 55–57, 59–65, 67, 68] controlled for patient characteristics and six [41, 55, 57, 58, 60, 65] controlled for health system or clinic factors (e.g., clinic site or specialty), and one [57] controlled for provider factors. Patient covariates included age (*n* = 12) [44, 55–57, 59–65, 67], gender (*n* = 6) [55–57, 63–65], insurance status (*n* = 6) [41, 44, 55, 56, 64, 67], race (*n* = 3) [59, 62, 65], ethnicity (*n* = 3) [59, 62, 65], marital status (*n* = 4) [41, 55, 56, 67], and chronic conditions (*n* = 4) [44, 62, 67, 68]. The only provider factors identified in the review were provider gender and professional status in one study [57]. Health system or clinic factors were included but only to control for random fixed effects for site of care or clinic location (*n* = 6) [41, 55, 57, 58, 60, 65].

Eleven studies conducted patient-level analysis [55, 56, 58–62, 64–66, 68]; of these, three [55, 59, 62] included the CG-CAHPS survey or used CG-CAHPS survey items, one [60] the Communication Assessment Tool (CAT) survey, one [61] the Parents’ Perception of Primary Care (P3C) survey, one [65] included the Interpersonal Processes of Care (IPC) survey, one [66] qualitative study used a study-specific focus group protocol, and the remaining four [56, 58, 64, 68] used a study-specific patient experience survey.

Four studies conducted provider-patient analysis [41, 44, 63, 67], including one [44] study that reviewed patient charts to document counseling on lifestyle changes; two studies that assessed provider communication, with one [41] using the Promoting Health Development Survey (PHDS) and another [63] the Interpersonal care (IPC) measures of communication; and one [67] study assessed patient-provider communication using a study-specific survey tool that assessed mental health needs in the past 12 months. Two studies conducted visit-level analysis [54, 57], including one [54] qualitative study of patients immediately after their appointment and one [57] study that coded recorded interactions between a patient and provider to identify patient-centered dialogue, and patient ratings of provider listening behavior.

### Examination of Evidence

Here, we review the evidence on the relationship between language concordance, the use of medical interpreters, and language preference and patient experience in primary care for Spanish-preferring patients.

The studies primarily used patient experience measures to assess associations (*n* = 13 studies) between either language concordance, use of interpreters, or language preference and their association with patient experience (Table 1). Two studies used patient experience measures to assess differences by subgroups, two studies were descriptive, and none evaluated interventions. Table 2 summarizes the associations of language concordance, use of interpreters, or Spanish language preference with better, worse, or no association with patient experience.

#### Associations Between Language Concordance and Patient Experience

The evidence on language concordance for Spanish-preferring patients in primary care shows a mix of null findings (4 studies) and associations with better patient experience (3 studies), in terms of receiving lifestyle counseling for diet and exercise [44], better interpersonal care processes (i.e., better clarify of communication, more elicited concerns, explained results, more respectful/compassionate provider interactions, more shared decision-making) [63], and better provider communication (i.e., more proactive and interaction communication) [65]. In addition, one study also examined health literacy in relation to language concordance and its association with patient experience and found significant associations between limited health literacy and worse patient experience (i.e., provider proactive and interactive communication) for Spanish-preferring patients seeing providers who speak Spanish (i.e., Spanish-concordant patients) [65], but not for Spanish-discordant patients. Furthermore, another study examined and found associations between providers’ self-reported cultural competence and better pediatric care experiences (i.e., more family-centered care, more helpfulness of care) [41].

#### Associations Between Use of Interpreters and Patient Experience

The evidence on the use of interpreters for Spanish-preferring patients in primary care is limited and a mix of studies finding better (two studies), worse (two studies), and no association (two studies) with patient experience. The use of interpreters was associated with better provider communication and staff courteousness (two studies), better access to care, and higher overall rating of care (one study each). Worse patient experience was found with the use of interpreters in studies that examined the content of interpreted conversations (two studies) finding significantly fewer statements conveyed by the interpreter (as compared to statements in the same encounter by the provider or by the patient) about medical information, medical questions, emotional statements, facilitation, lifestyle information/questions [57], and more content revisions, reductions, and omissions of provider-patient primary care discussions [54].

#### Associations Between Spanish-Language Preference and Patient Experience

Evidence on Spanish-language preference and its association with patient experience in primary care shows a mix of null findings (five studies) and worse patient experience (four studies). Spanish-language preference was associated with higher likelihood of wait times longer than an hour, difficulty getting information/advice over the phone, and no regular source of care or lack of continuity of care [56]; lower likelihood of being comfortable asking nurse questions, understanding nurses, and having medical problems resolved by end of the visit [58]; worse provider communication [60]; and for foreign-born (both Spanish-preferring and English preferring) parents and provider communication problems [59].

Online Resource 6 provides detailed study findings for patient experience measures grouped by setting (see Online Resource 6, which reviews relevant findings).

## Discussion

Our systematic review of 17 cross-sectional studies both confirmed that Spanish-preferring patients have worse patient experience in primary care [56, 58–60] and identified support for both promoting language concordant care [44, 63, 65] and for using formal, high-quality interpreters [55, 62] to improve patient experiences of primary care for Spanish-preferring patients. Studies indicated that a patient’s Spanish-language preference was associated with not having a regular source of care, lack of continuity of care, longer wait times, difficulty getting information and/or advice over the phone, being uncomfortable asking nurses questions, not understanding nurses, not having medical problems resolved by end of the visit, and having more provider communication problems. Language concordant primary care for Spanish-preferring patients improved several aspects of their experiences: Spanish-preferring patients who saw bilingual providers had better patient experiences in that they received lifestyle counseling on diet and exercise, raised more concerns, engaged in more shared decision-making, had results explained better, gained more clarification, had more proactive and interactive patient-provider communication, and more respectful and compassionate provider interactions. No evidence was found to address, however, the access and continuity issues identified for Spanish-preferring patients (i.e., not having a regular source of care, lack of continuity of care, longer wait times, difficulty getting information, and/or advice over the phone). Furthermore, when a Spanish-preferring patient was seen by a provider not fluent in Spanish (i.e., language-discordant care) and a formal interpreter was used, Spanish-preferring patients experienced better access to care, provider communication, and staff courteousness and rated their overall care higher. However, no evidence was found to address continuity of care, interactions with nurses, and having medical issues resolved at the end of the visit identified by Spanish-preferring patients. Additionally, when examining the content of interpreted conversations, studies generally found worse patient experiences [54, 57]. Interpreted conversations (compared to those with bilingual providers) conveyed less medical information, fewer medical questions and emotional statements, less facilitative statements, and information or questions about lifestyle. In addition, interpreted conversations also had more content revisions, reductions, and omissions. Interpreted conversations as a result are highly edited and different than bilingual conversations.

Evidence for this review on Spanish-language preference and its association with patient experience in primary care showed a mix of null findings (five studies) and worse patient experience (four studies). This supports the previous evidence (prior to 2005) that racial/ethnic and linguistic minorities tended to report worse care than did Whites and that linguistic minorities reported worse care than did racial and ethnic minorities [7–10].

Promoting bilingual providers and language concordant care is important for several reasons. First, language concordant care eliminates the time a provider spends engaging an interpreter, in theory allowing more provider time with the patient to address care needs. Second, language concordant care allows a more open interchange of information between patient and provider with less time needed for explanations related to language or the meaning of medical terms. However, the need for bilingual providers presents several challenges. It requires that medical groups and practices ensure that a provider is bilingual and qualified to conduct a medical visit in Spanish, so that the benefits in communication in the same language can occur. Relying on insufficient Spanish language fluency on the part of the provider is like relying on ad hoc interpreters, as both have high likelihood of poor or miscommunication, loss of information, and limited sharing and exchange. These challenges can potentially be overcome by targeted provider training, recruitment initiatives, or policy changes that encourage bilingualism in healthcare providers and staff. Furthermore, the pool of potential providers qualified as bilingual is often not adequate and may never be adequate, raising the need for additional strategies to support provider-patient communication.

Health literacy levels for Spanish-preferring patients vary, are generally lower than the general U.S. population [69, 70], and what drives it is not well understood [71]. Multiple systematic reviews have examined interventions to support health literacy, though most have focused on a specific chronic disease [72, 73]. Other reviews focus on individuals who are immigrants from Mexico or who are Spanish-preferring and identify limited evidence on the types of interventions effective to support health literacy [74, 75]. One education-based strategy has been rigorously studied and showed to improve both overall care and health literacy levels among Spanish-preferring patients, but that strategy focuses on the patient rather than the clinicians providing health information [76, 77]. As such, it is important not only to have literal translation of medical information, but translation that accounts for differences in health literacy and is both culturally competent and provided within the context of cultural humility (i.e., providers connecting to patients in a meaningful way through understanding their cultural background, needs, and preferences) [78]. Interventions, beyond translation, can be employed to address health literacy barriers and promote culturally competent, sensitive communication.

The strategy of providing professional, high-quality interpreters is challenging because the content of interpreted conversations is not equivalent to conversations with bilingual providers. Our review found evidence that much is “lost in translation,” meaning that during the translating process of converting one language to another some of the original meaning and intent is not conveyed or captured. Providing professional interpreters requires that medical groups and practices ensure that providers have access to high-quality translation (i.e., high-quality culturally competent and culturally sensitive interpreters who have medical interpretation training so that they understand medical conditions, medication, and medical terms that are used across multiple countries of origin). This starts with engaging with a high-quality interpretation service but also to conducting audits, soliciting feedback from providers and patients of the quality of interpretation, and the efficiency of setting up the interpretation service. Previous literature has shown that having culturally competent and culturally sensitive providers reduces disparities in care [79]. A review of systematic reviews found interventions to support provider cultural competency and sensitivity led to improvements in provider outcomes like knowledge, skills, and attitudes (i.e., through healthcare providers being capable and willing to work with their patients to ensure that that are both understanding and being understood) and weaker evidence on interventional effects on patient outcomes, including patient experience [80, 81].

Interpretation services could also allow medical contracts to only utilize interpreters with medical training and possibly provide the provider at each visit to be able to select the country of origin of the interpreter to best match that of the patient/family’s country of origin to maximize the use of similar and culturally competent language usage, also broadening interpreter training to more fully appreciate the importance of dialogue that captures patient-centered elements of communication and strategies to effectively convey these types of interchange and discussion. It is also important to recognize the role of active listening and non-verbal sensitivity in accurately identifying patient emotion as part of the interpretation process [57]. Toward this aim, future research should examine characteristics of interpreters (e.g., type of training, country of origin, ethnicity) in relation to the experiences of Spanish-preferring patients. Such findings may guide public health decision-makers toward specific quality improvement initiatives such as interpreter training in cultural sensitivity.

Providing bilingual written information and aides specific to medications and common conditions for both providers and interpreters can assist in facilitating ensured patient-provider understanding. Interventions are needed that train clinicians to customize information given to patients based on their health literacy level, and self-management support interventions, including group visits, telephone case management, and increased use of bilingual community health workers (or *promotoras*) [64] as navigators to improve quality of care. Provider training should emphasize a provider’s use of techniques such as “teach-back” and reducing the use of medical jargon to increase the chances of having more interactive communication with Spanish-preferring patients [65].

In addition to high-quality culturally competent interpreters, evidence from our review indicates that bilingual patient navigators hired at primary care clinics may be a strategy to reduce linguistic and cultural barriers to care [60]. These navigators can take many steps to improve patient experiences with care, including helping patients to make follow-up appointments, manage referrals (including with specialists who may not have Spanish-speaking front desk employees or call centers), and educate patients on their conditions and treatment options by spending more time talking to them after the visit. There also may be additional financial support available for primary care clinics to provide such navigation services, as the Centers for Medicare and Medicaid Services have proposed and are finalizing a rule that allows for reimbursement for navigation services for patients with high-risk conditions [82]. Moreover, policymakers ought to examine both the implementation of services aimed at mitigating the effects of language barriers as well as the implementation of existing federal and state legislation [56]. Policymakers could also support the implementation of language strategies by including reimbursement for navigation services and by addressing adherence to federal and state legislation regarding translation and the use of interpreters.

Furthermore, given the many null findings across both the strategies of language concordance care and using professional interpreters with patient experiences as well as the inherent challenges identified with both language concordant care and the use of interpreters, our review emphasizes that supportive language strategies are needed for all Spanish-preferring primary care patients whether they are cared for by qualified Spanish-speaking providers or need an interpreter during a primary care visit. Our review also points out that interpreter services are important to ensure patients and providers understand each other but is not sufficient if the translation is not of a high-quality and the time spent cannot cover all necessary content. The evidence identified one study that found that Spanish-preferring patients with bilingual providers also needed enough health literacy to effectively communicate about their health and understand the specifics of the healthcare interaction. On the other hand, another one study found that providers must have enough cultural competence, humility/sensitively to effectively communicate with a patient in a way that recognizes and accounts for a wide array of patient cultural backgrounds even if patients and the provider speak the same language.

Of note, all studies were cross-sectional (rather than randomized control, case control, cohort), which is typical of studies that are examining a cross-section of patients that seek care in the primary care setting and utilize patient experience measures as outcomes. Cross-sectional studies of examining changes in patient experience are also typically analyzed through the use of regression controlling for patient-, provider-, and clinic-level characteristics. The quality and utility of patient experience survey measures also varied. The use of objective measures of patient experience, such as Consumer Assessment of Healthcare Providers and Systems (CAHPS^®^) surveys which measure the frequency of actions and are the national standard for patient experience measurement, should be used more frequently in research, rather than measures with scales that capture patient perceptions of satisfaction that are subjective in nature. Thus, to identify evidence-based interventions that are generalizable across the various ambulatory care settings, research is needed with stronger designs such as randomized control studies and case control studies and rely on objectively measured outcomes of patient experience that are comparable (i.e., CAHPS measures).

### Limitations

This review has several potential limitations. Studies in which patient experience was not the focus or main outcome, and hence not mentioned in title or abstract, may have been missed. Also, there was substantial heterogeneity in the aspects of patient experience assessed and methods of patient experience data collection across our included studies. Therefore, we present a range of patient experiences for each strategy of interest. Our review, however, does identify 17 relevant articles that examine the use of medical interpreters in primary care for Spanish-preferring patients on patient experience, providing insights about medical interpreters and language concordance for Spanish-preferring patient experiences of care. However, much of the evidence about language concordant care or use of interpreters also identified null findings for patient experience. Importantly, however, our review underscores the importance of language concordant care, and the use of professional interpreters as did the reviews from over 15 years ago.

Research is needed that examines the influence that language concordance and interpreter use has on access and continuity of care for Spanish-preferring patients (i.e., not having a regular source of care, lack of continuity of care, longer wait times, difficulty getting information, and/or advice over the phone) as well as the need for interpreter service support for interactions with nurses in primary care, and ensuring that medical issues resolved at the end of the visit for all patients that are Spanish-preferring patients. Research is also needed to identify specific actions that can be integrated into the primary care workflow that support language needs. Such strategies need to be developed and tested so that healthcare organizations have available to them evidence-based strategies targeted at language support for all Spanish-preferring primary care patients whether they are cared for by qualified Spanish-speaking providers or need an interpreter. Additional research examining the lived experiences of Spanish-preferring patients and the primary care providers who care for them would be beneficial to understand the specific actions and strategies at the clinic-level, provider-level, interpreter-level, and patient-level could enrich how the use of language concordance and medical interpreters can support high-quality patient experiences for Spanish-preferring patients in the U.S.

## Conclusion

Past reviews identified that primary care with a bilingual provider or with the support of a formal interpreter improves overall care quality, but research was lacking on the associations with patient experience. Professional (versus ad hoc) interpreters in primary care were known to reduce communication errors, improve overall clinical care, but research was needed on interpreter quality and aspects of patient experience.

Our review found that Spanish-language preference by patients continues to be associated with worse patient experience. Evidence shows that language concordant provider-patient care is associated with better patient experience however may be challenged by Spanish-preferring patient’s limited health literacy or low levels of provider cultural competency. There are challenges in having an adequate pool of Spanish-qualified providers. Using professional interpreters is also associated with better patient experience but requires that medical groups and providers pay very close attention to whether their interpreter services are providing high-quality interpretation so that the use of interpreters does ensure comprehensive, effective patient-provider communication is achieved. Research is lacking in key areas of patient experience (such as continuity of care, ensuring reasons for visit are addressed, and translation for nurse communication conversations) related to language concordance and interpreter use for Spanish preferring patients. Policymakers, clinicians, leaders of ambulatory medical groups, and researchers need to re-engage in work that aims to understand, identify, and ultimately improve linguistic support for Spanish-preferring patients in primary care. As a result, supportive, evidence-based language strategies are needed to eliminate disparities that exist for all Spanish-preferring primary care patients whether they are cared for by qualified Spanish-speaking providers or need an interpreter. Research is needed to identify specific actions and evidence-based strategies that enrich how the use of language concordance and of medical interpreters can better support high-quality patient experiences for Spanish-preferring patients in the U.S.

## Supplementary Material

PRISMA

Online Resources2-6

## Figures and Tables

**Table 1:** Included studies’ main use of patient experience measures by language concordance, use of interpreters, and Spanish language preference grouped by setting

Use of patient experience	Language concordance (*N* = 7)	Use of interpreters (*N* = 6)	Spanish language preference (*N* = 5)	Other topic(s) studied (*N* = 3)

Associations to patient experience (*N* = 13)	Primary Care: Eamranond 2009	Primary Care: Moreno 2010 Roter 2020	Primary Care: Pippins 2007 Welty 2012	
	Pediatric Primary Care: Arauz Bourdreau 2010	Pediatric Primary Care: Flower 2017 Morales 2006	Pediatric Primary Care: Clemens-Cope 2007 Flower 2017	Pediatric Primary Care: Arauz Bourdreau 2010, Provider’s cultural competency
	Primary Care for Diabetes Patients: Detz 2014 Sudore 2009			Primary Care for Diabetes Patients: Sudore 2009, Fimited health literacy
	Primary Care focused on Mental Health: August 2011	Primary Care interfacing with Specialty Care: Song 2022		Primary Care interfacing with Specialty Care: Song 2022, Ophthalmology clinical adherence to recommended follow-up dilated fundus eye exam (DFE) (vs no DFE within one year) and ophthalmology visit attendance
Comparison of subgroups (*N* = 2)	Primary Care for Diabetes Patients: Rodriquez 2010		Pediatric Primary Care: Krugman 2009	
Descriptive (*N* = 2)	Primary Care for Diabetes Patients: Zamudio 2017	Primary Care: Aranguri 2006		

**Table 2:** Summary of association of language concordance, use of interpreters, and Spanish language preference and patient experience grouped by setting

	Language concordance (*N* = 7)	Use of interpreters (*N* = 6)	Spanish language preference + (*N* = 5)	Other topic(s) studied (*N* = 3)

**Better patient experiences (*N* = 6)**	Primary Care: +: Eamranond 2009 Primary Care for Diabetes Patients: +: Detz 2014, **+**: Sudore 2009	Primary Care: **+**: Moreno 2010 Pediatric Primary Care: +: Morales 2006		Pediatric Primary Care: +: Arauz Bourdreau 2010, **Provider’s cultural competency**
*Worse patient experience (N= 7)*		Primary Care: -: Roter 2020 -: Aranguri 2006	Primary Care: -: Pippins 2007 -: Welty 2012 Pediatric Primary Care: -: Clemens-Cope 2007 -: Flower 2017	Primary Care for Diabetes Patients: -: Sudore 2009, *Limited health literacy*
Null findings with patient experience (*N* = 12)	Primary Care: Null: Eamranond 2009 Pediatric Primary Care: Null: Arauz Bourdreau 2010 Primary Care for Diabetes Patients: Null: Rodriquez 2010 Primary Care focused on Mental Health: Null: August 2011	Pediatric Primary Care: Null: Flower 2017 Primary Care interfacing with Specialty Care: Null: Song 2022	Primary Care: Null: Pippins 2007 Null: Welty 2012 Pediatric Primary Care: Null: Clemens-Cope 2007 Null: Flower 2017 Null: Krugman 2009	Primary Care interfacing with Specialty Care: Null: Song 2022, Ophthalmology clinical adherence and ophthalmology visit attendance

+ with bold text indicates better patient care experience and (−) with *italics* indicates worse patient care experience. Regular font indicates no statistically significant differences or associations with patient care experiences. Zamudio et al. (2017) is not included in this table as the findings are not applicable given, they are descriptive qualitative themes

## Data Availability

Data sharing is not applicable for this article as no datasets were generated or analyzed during the current study.
