# Supplementary material for: Is Primary Care Patient Experience Associated with Provider-Patient Language Concordance and Use of Interpreters for Spanish-preferring Patients: A Systematic Literature Review
Source: J Racial Ethn Health Disparities. 2024 Mar 5;12(2):1170–83. doi: 10.1007/s40615-024-01951-z (PMC11374925; doi:10.1007/s40615-024-01951-z)
Supplement: Supplementary file 2 — Supplementary file2 (DOCX 108 KB) [file 40615_2024_1951_MOESM2_ESM.docx]

Is Primary Care Patient Experience Associated with Provider-Patient Language Concordance and Use of Interpreters for Spanish-preferring Patients: A Systematic Literature Review

Journal of Racial and Ethnic Health Disparities

Denise D. Quigley, Nabeel Qureshi, Zachary Predmore, Yareliz Diaz, Ron D. Hays

Corresponding Author: Denise Quigley, RAND Corporation, <quigley@rand.org>

**Online Resource 2. Search Strategy Terms**

| Terms Included | Key Words Used per Term |
| --- | --- |
| Primary care | ["primary care"[tiab] OR "ambulatory"[tiab] OR "internal medicine"[tiab] OR "family medicine"[tiab] OR "outpatient"[tiab] OR “walk-in clinic*”[tiab] OR FQHC*[tiab] OR "federally qualified health center*"[tiab] OR "health center*"[tiab] OR "community health"[tiab] OR "health clinic*"[tiab] OR “general practitioner*”[tiab] OR “primary care physician*”[tiab] OR “primary care nurs*”[tiab] OR "Primary Health Care"[Mesh] OR “Ambulatory Care Facilities”[Mesh] OR “Physicians, Primary Care”[Mesh]] |
| AND |  |
| Language | [interpreter*[tiab] OR interpretive[tiab] OR ((medical[tiab] OR professional[tiab] OR informal[tiab] OR formal[tiab]) AND interpret*[tiab]) OR (language*[tiab] AND (interpret*[tiab] OR translat*[tiab])) OR translator*[tiab] OR "language discord*"[tiab] OR "language concord*"[tiab] OR “language proficienc*”[tiab] OR "language assist*"[tiab] OR “language barrier*”[tiab] OR “communication barrier*”[tiab] OR "limited English proficienc*"[tiab] OR "LEP"[tiab] OR "language ability"[tiab] OR “language appropriate”[tiab] OR “language fluency”[tiab] OR “second language”[tiab] OR "non English speaking"[tiab] OR "non English prefer*"[tiab] OR "bilingual*"[tiab] OR "Spanish speaking"[tiab] OR "Spanish prefer*"[tiab] OR "Translating"[Mesh] OR "Communication Barriers"[Mesh:noExp] OR "Limited English Proficiency"[Mesh] OR “Hispanic or Latino”[MAJR]] |
| AND |  |
| Patient experience | ["patient experience*"[tiab] OR "patient-center*"[tiab] OR "patient center*"[tiab] OR "patient satisfaction"[tiab] OR "patient preference*"[tiab] OR "patient adherence*"[tiab] OR "patient compliance*"[tiab] OR “health care utiliz*”[tiab] OR "access to care"[tiab] OR "access to health*"[tiab] OR "access to med*"[tiab] OR "office staff*"[tiab] OR "reception desk*"[tiab] OR "front office"[tiab] OR "call center*"[tiab] OR "scheduling"[tiab] OR "referral*"[tiab] OR referring[tiab] OR "timeliness"[tiab] OR timely[tiab] OR "no-show*"[tiab] OR "canceled appointment*"[tiab] OR "missed appointment*"[tiab] OR "continuity of care"[tiab] OR "CAHPS"[tiab] OR "consumer assessment"[tiab] OR "patient survey*"[tiab] OR "Ganey"[tiab] OR (("test result*"[tiab] OR "lab result*"[tiab] OR "laboratory result*"[tiab]) AND ("communicat*"[tiab] OR explain*[tiab] OR explanation*[tiab])) OR “language preference*”[tiab] OR “language access*”[tiab] OR “communication preference*”[tiab] OR “appointment adherence”[tiab] OR "Appointments and Schedules"[Mesh:NoExp] OR “Language”[MAJR] OR "Patient Satisfaction"[Mesh] OR "Patient Preference"[Mesh] OR "Continuity of Patient Care"[Mesh:noExp] OR "Health Knowledge, Attitudes, Practice"[MAJR] OR "Access to Primary Care"[MAJR] OR "Health Services Accessibility"[MAJR] OR “Patient Acceptance of Health Care”[MAJR]] |
| Filters: | Humans  English  US-based studies  Adult: 19+ years  January 2005 – January 31, 2023  Excluded: Commentaries/editorials |
| Databases: | PubMed/MEDLINE  CINAHL (EBSCOhost)  APA PsycInfo (EBSCOhost)  COCHRANE  Web of Science |

Is Primary Care Patient Experience Associated with Provider-Patient Language Concordance and Use of Interpreters for Spanish-preferring Patients: A Systematic Literature Review

Journal of Racial and Ethnic Health Disparities

Denise D. Quigley, Nabeel Qureshi, Zachary Predmore, Yareliz Diaz, Ron D. Hays

Corresponding Author: Denise Quigley, RAND Corporation, <quigley@rand.org>

**Online Resource 3. Quality Ratings of Included Articles using the Joanna Briggs Institute (JBI) Critical Appraisal Tool for Analytical Cross-Sectional Studies**

| **Cross-Sectional Studies** | | | | | | | | | |
| --- | --- | --- | --- | --- | --- | --- | --- | --- | --- |
| **Study** | **Selection** | | **Exposure** | | **Analysis** | | | | **JBI Total** |
| *Author Year* | *Inclusion Criteria Defined* | *Subjects and Setting Described* | *Exposure Measured Reliably* | *Objective Criteria for Condition* | *Confounding Factors identified* | *Confounding Factors Addressed* | *Outcomes Measured Reliably* | *Appropriate Statistics Used* | Max=8 |
| Arauz Boudreau 2010 | Yes | Yes | Yes | Yes | Yes^P,MD,C^ | Yes^P,MD,C^ | Yes | Yes | 8 |
| August 2011 | Yes | Yes | Yes | Yes | Yes^P^ | Yes^P^ | Yes | Yes | 8 |
| Clemans-Cope 2007 | Yes | Yes | Yes | Yes | Yes^P,C^ | Yes^P,C^ | Yes | Yes | 8 |
| Detz 2014 | Yes | Yes | Yes | Yes | Yes^P^ | Yes^P^ | Yes | Yes | 8 |
| Eamranond 2009 | Yes | Yes | Yes | Yes | Yes^P^ | Yes^P^ | Yes | Yes | 8 |
| Flower 2017 | Yes | Yes | Yes | Yes | Yes^P,C^ | Yes^P,C^ | Yes | Yes | 8 |
| Krugman 2009 | Yes | Yes | Yes | Yes | Yes^P,C^ | Yes^P,C^ | Yes | Yes | 8 |
| Morales 2006 | Yes | Yes | Yes | Yes | Yes^P^ | Yes^P^ | Yes | Yes | 8 |
| Moreno 2010 | Yes | Yes | Yes | Yes | Yes^P,C^ | Yes^P,C^ | Yes | Yes | 8 |
| Pippins 2007 | Yes | Yes | Yes | Yes | Yes^P^ | Yes^P^ | Yes | Yes | 8 |
| Rodriguez 2010 | Yes | Yes | Yes | Yes | Yes^P^ | Yes^P^ | Yes | Yes | 8 |
| Roter 2020 | Yes | Yes | Yes | Yes | Yes^P,MD,C^ | Yes^P,MD,C^ | Yes | Yes | 8 |
| Soh 2022 | Yes | Yes | Yes | Yes | Yes^P,MD^ | Yes^P,MD^ | No | Yes | 7 |
| Song 2022 | Yes | Yes | Yes | Yes | Yes^P^ | Yes^P^ | Yes | Yes | 8 |
| Sudore 2009 | Yes | Yes | Yes | Yes | Yes^P,MD,C^ | Yes^P,MD,C^ | Yes | Yes | 8 |
| Welty 2012 | Yes | Yes | Yes | Yes | Yes^P,C^ | Yes^P,C^ | Yes | Yes | 8 |
| Winkler 2022 | Yes | Yes | Yes | Yes | Yes^P^ | Yes^P^ | Yes | No | 7 |

NOTE: We dropped 5 articles for poor quality as they scored a 5 or below out of 8 on the JBI. These included four cross-sectional studies and one cohort study. These articles did not report information about the sample selection and had inadequate analysis techniques, including not addressing potential confounding factors and/or using t-tests without controls. We have provided additional information about confounding factors. If confounding factors were identified or addressed at the patient (P), provider (MD), or clinic (C) level, we have added superscripts to the “Yes” indicating the level.

Is Primary Care Patient Experience Associated with Provider-Patient Language Concordance and Use of Interpreters for Spanish-preferring Patients: A Systematic Literature Review

Journal of Racial and Ethnic Health Disparities

Denise D. Quigley, Nabeel Qureshi, Zachary Predmore, Yareliz Diaz, Ron D. Hays

Corresponding Author: Denise Quigley, RAND Corporation, <quigley@rand.org>

**Online Resource 4. Quality Ratings of Included Qualitative Articles using the Joanna Briggs Institute (JBI) Critical Appraisal Tool for Qualitative Studies**

| **Qualitative Studies** | | | | | | | | | | | |
| --- | --- | --- | --- | --- | --- | --- | --- | --- | --- | --- | --- |
| **Study** | **Congruity of Methods and Analysis** | | | | | **Researcher Influence** | | | **Ethics** | **Conclusions** | **JBI Total** |
| *Author Year* | Philosophical perspective | Research methodology | Methods | Analysis of data | Interpretation of results | Locating the researcher culturally | Influence of the researcher | Participant voices represented | Ethical research | Conclusions flow from analysis | Max=10 |
| Aranguri 2006 | Yes | Yes | Yes | Yes | Yes | No | No | Yes | Yes | Yes | 8 |
| Zamudio 2017 | Yes | Yes | Yes | Yes | Yes | No | No | Yes | Yes | Yes | 8 |

Is Primary Care Patient Experience Associated with Provider-Patient Language Concordance and Use of Interpreters for Spanish-preferring Patients: A Systematic Literature Review

Journal of Racial and Ethnic Health Disparities

Denise D. Quigley, Nabeel Qureshi, Zachary Predmore, Yareliz Diaz, Ron D. Hays

Corresponding Author: Denise Quigley, RAND Corporation, <quigley@rand.org>

**Online Resource 5. Main Topic, Methods, Population, Measures for Included Studies, By Setting**

| **Main Study Topic** | **Design and**  **Statistical Approach** | **Population Characteristics and Sample Size** | **Measures and Data Collection Timeframe** | **Other Outcomes** |
| --- | --- | --- | --- | --- |
| **Primary Care (n=6)** | | | | |
| **Aranguri 2006** | | | | |
| - Descriptive - Content of provider-patient primary care discussions *using an interpreter* | - Qualitative - Patient interviews in Spanish immediately after appointment - Visit level analysis. - Transcript analysis of questions asked and answered during primary care visit | - 16 patients having dyslipidemia and 9 physicians (including 1 bilingual physician) from primary care clinics in New York, California, Texas, and Florida, of which 13 patients used an informal interpreter (family member n=2, nursing/office staff n=11) and 3 patients spoke Spanish with the bilingual physician | - Interpreters included informal interpreters (family members, nursing or office staff) - Time speaking (by physician, patient, interpreter), any loss of semantic information of the patient, and content omissions, revisions and reductions. - Dates of data collection not reported | None |
| **Eamranond 2009** | | | | |
| - Associations - Relationship between *language concordance* and lifestyle counseling | - Cross-sectional - Retrospective chart review covering 3 years prior to and including the most recent PCP visit. - Patient-provider level analysis - Multivariate logistic regression models, controlling for age, sex, insurance status, number of PCP visits, and Charlson comorbidity index | - 306 patients receiving care from two primary care practices (1 academic hospital-based practice with 4 Spanish-speaking attending physicians and 44 non-Spanish speaking attending physicians and 1 community-based ambulatory care center with 2 Spanish-speaking attending physicians and 5 non-Spanish speaking physicians), of which 205 were language-concordant and 101 were language-discordant | - Language concordance was defined by whether a PCP could converse fluently in Spanish. - Documented lifestyle counseling on exercise, diet, and smoking. Counseling on diet and exercise had to be directly related to overall cardiovascular health, not for a specific condition. Discussion of smoking was based on whether PCPs documented patient smoking status and/or had other discussions on smoking cessation. - Patient charts from January 1, 2000, to June 30, 2006 | None |
| **Moreno 2010** | | | | |
| - Associations - Relationship between *need and use of interpreters* and patient experience | - Cross-sectional - CAHPS 2.0 surveys (via computer-assisted telephone interviews (CATI) with bilingual interviewers) of patient experience of care (including interpreters) in the last 6 months. - Patient-level analysis - Unadjusted and adjusted multivariate regression, controlling for age, gender, marital status, education, income, insurance, self-reported health status, survey wave/year, and random effects for site | - 1,590 Spanish-preferring Latino adults from 8 outpatient sites across the United States (Alabama, Nebraska, Pennsylvania, Rhode Island, South Carolina, Texas, California, and Washington) in The Hablamos Juntos – Together We Speak (HJ) project | - Need and use of interpreters defined by responses to two questions about interpreters (i.e., whether you needed an interpreter, and if so, how frequently they had interpreters available for use (always, usually, sometimes, never)), creating three groups: Did not need interpreter; Needed interpreter, always available; and Needed interpreter, not always available. - Type of interpreters not specified. - CAHPS measures of provider communication (4 items; listening carefully, explaining things in ways a patient could understand, showing respect for what patients have to say, spending enough time), office staff helpfulness (2 items; focused on being courteous and respectful and being helpful) and overall rating of care. - Surveys administered in 2003 and 2006 | None |
| **Pippins 2007** | | | | |
| - Associations - Relationship between *Spanish language preference* and primary care experiences | - Cross-sectional - National household survey of adults, i.e., National Latino and Asian American Study (NLAAS), restricted to those self-identified as Latinos (completed in English or Spanish) about language proficiency and primary care experiences. - Patient-level analysis - Multivariate logistic regression, controlling for Latino ethnic subgroup, age, gender, marital status, education, insurance status, number of comorbidities, 30-day functioning and region | - 1,792 Latino adults with health insurance receiving primary care | - English language proficiency measured as a binary variable (poor/fair versus good/excellent) and based on response to, “How well do you speak English?” - Patient experience measures of wait times longer than an hour, difficulty getting information/advice over the phone, difficulty getting appointment over the phone and no regular source of care (or lack of continuity of care - Surveys administered May 2002 through November 2003 | None |
| **Roter 2020** | | | | |
| - Associations - Content of provider-patient primary care discussions *using an interpreter* - Relationship between *use of interpreter* and patient experience | - Cross-sectional - Audio-recorded primary care visits followed by a post-visit in-person patient survey (in patient’s preferred language) - Visit-level analysis using the Roter interaction analysis system (RAIS), which assigns one of 37 mutually exclusive and exhaustive codes to every statement and assigns a speaker designation (provider, interpreter-as-provider, patient, and interpreter-as-patient) - Multivariate mixed negative binomial regression, controlling for patient-centeredness ratio (provider and patient versus interpreter-as-provider and interpreter-as-patient), patient language, speaker-by-language interaction, visit length, patient age, gender, education, co-morbidity count, whether the visit was with the patient's regular provider, and whether the patient was accompanied by a caregiver, provider gender, and professional status (attending, resident) as well as random effects for providers and clinic visits. | - 55 patients (27 Cantonese, 17 Mandarin and 11 Spanish-speaking primary care patients) in a large, urban academic outpatient primary care clinic with 31 physicians who used professional interpreter services during a recent audio-recorded visit | - Interpreters were only professional interpreters. - Patient experience measured by patient-centered dialogue and rating of interpreter listening. - Patient-centered dialogue included measures of: 1) medical information (including codes reflecting medical information, therapeutic regimen information, *medical/treatment counseling statements); 2) medical questions (including open and closed questions about the medical condition and treatment); 3) psychosocial/lifestyle information (psychosocial information, lifestyle information and *psychosocial/ lifestyle counseling); 4) psychosocial/ lifestyle questions (open and close-ended questions about psychosocial and lifestyle topics); 5) emotional statements (concern, reassurance, empathy, legitimation, partnering, self-disclosure*); and 6) partnership facilitation statements (e.g., asking for understanding, asking for reassurance, cues of interest, checks for understanding, asking for opinion*, *asking for permission to proceed). Asterisks denote provider or interpreter-as-provider only codes. - Also, patients were asked to rate interpreters’ listening behavior (i.e., “How was the interpreter at listening to what you had to say?” - Dates of data collection not reported | None |
| **Welty 2012** | | | | |
| - Associations - Relationship between *language preference* and patient experience | - Cross-sectional - Survey of patient experience at current visit (completed by paper at time of visit) - Patient-level analysis - Multivariate logistic regression, controlling for reason for visit, clinic location, and time spent in the waiting room | - 1,344 patients at seven primary care clinic sites within Jefferson County Department of Health in Alabama including adult and parent/child visits of which 253 (19%) were Spanish-preferring patients and 1,091 (81.2%) English-preferring patients | - Interpreter services measure (on Spanish survey only) asked whether interpreter was friendly and courteous. - Type of interpreters not specified. - Patient experience measures of ease of getting an appointment, comfort asking provider questions, comfort asking nurse questions, understanding of health information/instructions by provider, understanding of health information/instructions by nurse, medical problem resolved before leaving, courtesy of front desk staff, courtesy of medical staff, and overall rating of care; all dichotomized to reflect agreement or disagreement. - Surveys administered March 19 to April 19, 2008 | None |
| **Pediatric Primary Care (n=5)** | | | | |
| **Arauz Boudreau 2010** | | | | |
| - Associations - Relationship between *language concordance* and provider’s cultural competency and parent’s experience of child’s care | - Cross-sectional - Surveys about pediatric primary care in the last 12 months via mail in both English and Spanish - Pediatric primary care provider surveys including self-rated language ability and cultural competency. - Parent-provider level analysis - Unadjusted and adjusted multivariate linear regression, controlling for parents’ education, marital status, years in US, insurance status, interpreter use, and site of care | - 462 Latino parents with child well-care experiences at three community health centers - 22 pediatric primary care providers (PCP) | - Language concordance defined as parents reporting that they speak the same language at home as they do with their child’s PCP. - Type of interpreters not specified. - Care experience measures from the Promoting Health Development Survey (PHDS)[83] of provider communication (including anticipatory guidance and parental education topics discussed; anticipatory guidance and parental education needs met; assessment of family risk factors), family-centered care; and helpfulness of care provided. - Surveys administered January through March 2006 | - Self-rated provider cultural competency measures: perceived effectiveness in caring for Latino patients; perceived understanding of health-related cultural beliefs of Spanish-speaking patients |
| **Clemans-Cope 2007** | | | | |
| - Associations - Relationship between characteristics of low-income families (including *language preference/nativity*) and parent’s experience of provider communication problems with child’s care | - Cross-sectional - National household survey, National Survey of America’s Families, which oversamples low-income population. - Patient-level analysis - Multivariate linear probability regression, controlling for child’s race and ethnicity, and dummy variables for four categories of child’s age, three categories of family structure, three or more children in household, thirteen oversampled states, and survey year | - 24,485 children (0-17 years old) from low-income families of which 1,632 had Spanish-preferring parents | - Parent’s nativity and language preference categories were US-born English-preferring, US-born Spanish-preferring, foreign-born English-preferring, foreign-born Spanish-preferring. - Provider communication problems defined as provider does not listen or explain things carefully (measured by parent reports of never or sometimes (vs usually or always) on the two CAHPS items: “In the last 12 months, how often have your family’s providers or other health professionals listened to you carefully? and how often have your family’s providers or other health professionals explained things in a way you could understand?” - Surveys administered in 1999 and 2002 | None |
| **Flower 2017** | | | | |
| - Associations - Relationship between *language preference* and *interpreter use* and communication about child’s care | - Cross-sectional - Survey about pediatric 2-month well visits in English and Spanish - Post-visit provider survey about in-person interpreter use and language of the visit. - Patient-level analysis - Multivariate generalized linear regression modeling, controlling for parent age, education, income, health literacy, and clinic site. - Models run also for only visits among Spanish-preferring patients with and without interpreter | - 862 parents of children at 2-month well visits at 4 pediatric academic medical center clinics in New York, Tennessee, North Carolina, and Florida of which 303 Spanish-preferring Latinos, 127 English-preferring Latinos, and 432 English-preferring non-Latinos | - Language and ethnicity were combined and defined as Spanish-preferring Latino, English-preferring Latino, and English-preferring non-Latino. - Type of interpreter not specified. - Communication Assessment Tool (CAT)[84] measures of provider communication (14 items) and staff communication (1 item) - Surveys administered April 2010 through December 2014 | None |
| **Krugman 2009** | | | | |
| - Comparison of subgroups - Pediatric care experiences by *language preference* | - Cross-sectional - Surveys about pediatric primary care in both English and Spanish - Clinics provided information on interpretive services, including bilingual non-physician staff, bilingual physicians, and professional in-person/onsite or phone interpreters. - Patient-level analysis - Multivariate linear regression controlling for child’s age, race, parent’s education, number of visits, ability to identify provider, and random effects | - 2,122 parents of children receiving care in 19 pediatric resident continuity practices (7 East coast, 8 West coast, and 4 Midwest) in the Continuity Research Network (CORNET) of which 490 (23%) were Spanish-preferring and 1,632 (77%) were English-preferring | - Parents’ Perception of Primary Care (P3C) measures[85] of access, longitudinal continuity, comprehensiveness, coordination, communication, and contextual knowledge - Interpreters were professional interpreters (in-person/onsite or phone) - Surveys administered May through June 2004 | None |
| **Morales 2006** | | | | |
| - Associations - Relationship between *use of interpreter* and patient experience | - Cross-sectional - CAHPS 2.0 surveys (via mail and phone) of parents of children enrolled in the California State Children’s Health Insurance Program (S-CHIP) - Patient-level analysis - Multivariate regression model, controlling for parent age, parent education, child age, child race/ethnicity, parent rating of child’s health, indicator for child with a chronic condition, and survey language and clustering within health plans | - 26,671 parents of children in S-CHIP, which includes 26 health plans, of which 47% were Spanish-preferring, 42% English-preferring, and 11% were Asian preferring (Cantonese, Korean, or Vietnamese) | - Interpreter use defined by whether in the past 6 months the parent needed an interpreter and how frequently they used an interpreter. - Interpreters were trained, professional interpreters. - CAHPS measures of provider communication (4 items), access to care (4 items), timeliness of care (4 items) and staff helpfulness (2 items) summarized into groupings: provider and office staff communication (six items) and access to care (eight items). - Surveys administered in 2000 and 2001 | None |
| **Primary Care for Adult Diabetes Patients (n=4)** | | | | |
| **Detz 2014** | | | | |
| - Associations - Relationship between *language concordance* and interpersonal care | - Cross-sectional - Survey interview (via phone) of Latino adults with type-2 diabetes about experiences with primary care provider in last 12 months linked to clinical data and diabetes registry. - Survey of physicians practicing at FQHC including self-rated language ability - Patient-provider level analysis - Multivariate linear regression, controlling for age, gender, education, and income | - 248 Latino patients with diabetes receiving care from a FQHC with 20 primary care clinics and 31 physicians in two rural counties in California, of which 120 are language-discordant and 128 language-concordant. | - Language concordance defined as Spanish-speaking patients whose physicians self-rated as fluent in Spanish (excellent or good self-reported fluency) - Interpersonal processes of care[86] (IPC) measures included clarity of communication (two items), elicited concerns and responded (three items), explained results (two items); decision-making and decided together (two items)); and interpersonal style including compassionate and respectful (three items) and discriminated (two items) - Surveys administered July 2009 through January 2010 | - Diabetes self-care measures: participation in foot care, healthy eating, exercise, self-monitoring, medication adherence |
| **Rodriguez 2010** | | | | |
| - Comparison of subgroups - *Language concordance* and patient experience by usual care sites | - Cross-sectional - National interview survey (via phone) of Latino adults, i.e., 2007 Pew Hispanic Center/RWFJ Latino Health Survey, about quality of care in the last 12 months - Patient-level analysis - Chi-squared and t-tests of patient’s usual care site and patient sociodemographic characteristics - Unadjusted and adjusted ordinary least squares linear regression, controlling for age, gender, acculturation, spirituality, income, education, self-rated physical health, depression, insurance/utilization status, use of folk healer, language discordance and region. - Blinder-Oaxaca decomposition method was used to determine drivers of differences in patient experiences between community health centers and private physician practices | - 583 Latino diabetic patients of which 423 were interviewed in Spanish and 160 in English - Usual care sites included community health centers (n=237) and private physician offices (n=246), or no source of usual care (n=100) | - Provider-patient language concordance measured by responses to patient’s ability to carry on a conversation in English, both understanding and speaking, and the language reported as generally being spoken during most medical appointments. If a patient endorsed not speaking English very well and   had most appointments in English, they were categorized as receiving language discordant care, whereas if most appointments were in Spanish, they were categorized as receiving language concordant care.   - Patient experience was measured by an overall rating of care received in last 12 months and problematic care experiences, including three measures of confusion about information given, frustration by lack of information or inability to find out information wanted to know, and reassured about capacity to manage own health. - Surveys conducted July 16, 2007, through September 23, 2007 | None |
| **Sudore 2009** | | | | |
| - Associations - Relationship between *language concordance* (and *health literacy)* and provider communication | - Cross-sectional - Patient interview survey (via phone) about provider communication in the last 6 months including short form Test of Functional Health Literacy (HL) in Adults (in English or Spanish) - Patient-level analysis - Multivariate regression models including predictor variables of health literacy, language concordance, and an interaction term, controlling for patient age, race, gender, education, site of care, and clustering by physician. | - 771 adult patients with diabetes and/or cardiac disease, who have a primary care provider or cardiologist, and had an outpatient visit with that provider in the last 6 months | - Health literacy was[87] as limited HL with scores <=22/36 and adequate HL as scores >22/36 - Language categories included English-preferring patients with English-speaking providers, Spanish-preferring patients with Spanish-speaking providers (Spanish language-concordant), and Spanish-preferring patients with English-speaking providers (Spanish language-discordant). - Provider communication measures from the Interpersonal Processes of Care (IPC) instrument in Diverse Populations)[88-90] of receptive, proactive, and interactive communication, based on questions, ‘‘In the past 6 months, how often did you feel confused about what was going on with your medical care because your provider did not explain things well?’’ (receptive); ‘‘. . . how often did your provider give you enough time to say what you thought was important?’’ (proactive); and ‘‘. . . how often did your provider ask if you might have any problems doing the recommended treatment?’’ (interactive). - Surveys administered from 2000 through 2005. | None |
| **Zamudio 2017** | | | | |
| - Descriptive - Relationships of culture and *language concordance* on patient experience | - Qualitative - Conduct 2 focus groups (1 male, 1 female) with patients who received care from English-speaking providers (language discordant) and 2 focus groups (1 male, 1 female) with patients who received care from Spanish-speaking providers (language concordant) - Patient-level analysis - Content analysis | - 36 primary care patients with poorly controlled diabetes (i.e., elevated HbA1c ) receiving care from Kaiser Permanente Northern California (KPNC) of which all were non-US born Spanish-preferring adults (17 women, 19 men) | - Focus group discussions focused on visit preparation, provider communication, and role of other care team members with a focus language concordance or discordance. - Dates of focus groups not reported | None |
| **Primary Care focused on Mental and Behavioral Health (n=1)** | | | | |
| **August 2011** | | | | |
| - Associations - Relationship of *language concordance* and patient-provider discussion of mental health needs | - Cross-sectional - Patient interview survey (via phone) from the California Health Interview Survey (CHIS) about mental health needs in the past 12 months and the discussion of those needs with a physician at their last visit in English, Spanish, Chinese (Mandarin and Cantonese), Vietnamese, and Korean - Patient-provider level analysis - Multivariate logistic regression, controlling for age, sex, marital status, nativity, insurance status, education and mental health status | - 2,960 adults aged 55+ who had seen a primary care provider in the last 2 years of which 1,46 were Latino and 1,614 were Asian Pacific Islander | - Language concordance defined by what language patient spoke at home, spoke with their provider, and whether they needed help in understanding their provider, creating three groups: English-language concordant, other language concordant, language discordant. - Patient-provider communication measures[29, 91] about mental health needs in the past 12 months and the discussion of those needs with a physician at their last visit - Surveys administered July 2007 through March 2008 | None |
| **Primary Care Interfacing with Specialty Care (n=1)** | | | | |
| **Song 2022** | | | | |
| - Associations - Relationship between *use of interpreter* and specialist referral | - Cross-sectional - Retrospective chart review - Patient-level analysis - Bivariate analyses with generalized estimating equations - Multivariate logistic regression, controlling for ethnicity, need for interpreter services, diabetic nephropathy, diabetic neuropathy, and glomerular filtration rate (GFR). | - 1,107 uninsured and underinsured adult patients who underwent a teleretinal imaging (TRI) at a Federally Qualified Health Center in Durham, North Carolina, with 722 Hispanic and 623 who reported needing an interpreter | - Patient experience measures were whether a patient received a specialist referral from the FQHC provider for a follow-up dilated fundus eye exam (DFE) by an ophthalmologist (vs repeat TRI) - Interpreter type was unspecified. - Patient charts from January 2015 through September 2019 | - Ophthalmology clinical adherence to recommended follow-up DFE (vs no DFE within one year) and ophthalmology visit attendance |

**References for Online Resource 5:**

83. The Commonwealth Fund. The Promoting Healthy Development Survey (PHDS) 2006 [Available from: <https://www.commonwealthfund.org/sites/default/files/documents/___media_files_resources_2006_the_promoting_healthy_development_survey__implementation_guidelines_combined_apps_pdf.pdf>. Accessed October 12, 2006.

84. Makoul G, Krupat E, Chang CH. Measuring patient views of physician communication skills: development and testing of the Communication Assessment Tool. Patient Educ Couns. 2007;67:333-42. doi:10.1016/j.pec.2007.05.005

85. Seid M, Varni JW, Bermudez LO, et al. Parents' perceptions of primary care: Measuring parents' experiences of pediatric primary care quality. Pediatrics. 2001;108:264-70. doi:10.1542/peds.108.2.264

86. Stewart AL, Napoles-Springer AM, Gregorich SE, et al. Interpersonal processes of care survey: patient-reported measures for diverse groups. Health Serv Res. 2007;42:1235-56. doi:10.1111/j.1475-6773.2006.00637.x

87. Seligman HK, Wang FF, Palacios JL, et al. Physician notification of their diabetes patients' limited health literacy. A randomized, controlled trial. J Gen Intern Med. 2005;20:1001-7. doi:10.1111/j.1525-1497.2005.00189.x

88. Piette JD, Schillinger D, Potter MB, et al. Dimensions of patient-provider communication and diabetes self-care in an ethnically diverse population. J Gen Intern Med. 2003;18:624-33. doi:10.1046/j.1525-1497.2003.31968.x

89. Stewart AL, Napoles-Springer A, Perez-Stable EJ. Interpersonal processes of care in diverse populations. Milbank Q. 1999;77:305-39, 274. doi:10.1111/1468-0009.00138

90. Stilling D, Bindman A, Wang F, et al. Functional health literacy and the quality of physician–patient communication among diabetes patients. Patient Educ Couns. 2004;52:315–23.

91. Jacobs E, Chen AH, Karliner LS, et al. The need for more research on language barriers in health care: A proposed research agenda. Milbank Q. 2006;84:111-33. doi:10.1111/j.1468-0009.2006.00440.x

Is Primary Care Patient Experience Associated with Provider-Patient Language Concordance and Use of Interpreters for Spanish-preferring Patients: A Systematic Literature Review

Journal of Racial and Ethnic Health Disparities

Denise D. Quigley, Nabeel Qureshi, Zachary Predmore, Yareliz Diaz, Ron D. Hays

Corresponding Author: Denise Quigley, RAND Corporation, <quigley@rand.org>

**Online Resource 6. Results for Patient Experience, By Setting**

| Study | Results for Patient Experience Measures and Ratings |
| --- | --- |
|  | **Primary Care** |
| Aranguri 2006 | **Worse patient experience (i.e., more content revisions, reductions and omissions of provider-patient primary care discussions) found *when using an interpreter* (compared to being conducted solely in Spanish*)*.**   - Speech was significantly reduced and revised by the informal interpreter, resulting in alterations of linguistic features such as content, meaning, reinforcement/validation, repetition, and affect. Visits that included an interpreter had virtually no rapport-building ‘‘small talk,’’ which typically enables the physician to gain comprehensive patient history, learn clinically relevant information, and increase emotional engagement in treatment. - Time speaking (by physician, patient, interpreter) showed that physicians spoke 49% of the words spoken in the visit, patients spoke 24%, and interpreters spoke 27%; this means that both physicians and patients said more than is being translated, and in the case of physicians, much more. This analysis shows unambiguously that not everything that is said is being interpreted. - Content omissions (leaving out important information) was observed most frequently when the interpreter reduced phrases with multiple semantic elements to one ‘‘key’’ element. - Content revisions (changing important information) appeared often less an attempt to simplify a multipart utterance to its key component (as with content omissions), and more a simple difficulty in conveying the semantic content of the utterance with precision. - Content reductions (synthesizing long passages of talk) appeared more often when physicians or patients had produced exceptionally long stretches of talk without providing a break for interpreting. In these cases, responsibility may be more with the primary interlocutors (physician or patient) than with the interpreter, who cannot be expected to retain entire paragraphs at a time and then report them verbatim. - Any loss of semantic information of the patient (was content not raised) was evident when comparing content to interpreted visits and visits conducted solely in Spanish. Most striking was the near-categorical lack, in interpreted visits, of what is frequently referred to as ‘‘small talk,’’ that is, socially oriented talk that is designed to further relationships rather than establish medical facts. |
| Eamranond 2009 | **Association between *language concordance* and better patient experience (i.e., receiving lifestyle counseling for diet and exercise); but no association between *language concordance* and counseling on smoking.**   - Significant association between provider-patient language concordance and higher likelihood to have **patient counseling on diet** (odds ratio (OR) = 2.2, CI 1.3–3.7, p-value=0.01) and **on physical activity** (OR = 2.3, CI 1.4–3.8, p-value=0.01) as compared to patients with language-discordant providers, controlling for age, sex, insurance status, number of primary care visits, and comorbidity score. - No significant difference found between provider-patient language-concordance with regard to discussion of **smoking** (OR = 1.3, CI 0.8–2.1). |
| Moreno 2010 | **Association between the *need and use of interpreters* and better patient experiences, i.e., better CAHPS provider communication, staff courteousness and overall rating of care.**   - Positive, significant association between *Needed interpreter, always available* and **overall rating of care** [3.65 point increase (Standard error (SE)=1.19; p-value<0.01)], **provider communication** [6.04 point increase (SE=1.47; p-value <0.001)] and **staff courteousness** [5.29 point increase (SE=1.83; p-value <0.001)] as compared to *Did not need interpreter*, controlling for age, gender, marital status, education, income, insurance, self-reported health status, survey wave/year, and random effects for site. |
| Pippins 2007 | **Association between *Spanish language preference* (i.e., resulting from poor/fair English language proficiency) and worse patient experiences, i.e., higher likelihood of wait times longer than an hour, difficulty getting information/advice over the phone, and no regular source of care or lack of continuity of care. No Associations between *Spanish language preference* and difficulty getting appointments scheduled** **by phone**.   - Significant association found between *Spanish language preference* *(i.e., poor/fair English language proficiency)* and higher likelihood to report **wait times of longer than an hour** (OR=1.88; 95%CI 1.34-2.64), **difficulty getting information or medical advice over the phone** (OR=1.76, 95% CI 1.24-2.46), **and no regular source of care or lack of continuity of care** (OR=2.20 95%CI 1.60-3.02) when compared to those with good/excellent English language proficiency. - No association was found between *Spanish language preference* and **difficulty getting appointments scheduled** **by phone**. |
| Roter 2020 | **Association between *interpreter mediation* and worse patient experience (i.e., corresponding statements made by physician and interpreter and by patient and interpreter).**   - Significant association between a higher amount of provider statements (compared to interpreter-as-provider statements) about **medical information** (Factor Change (FC) 1.27, p-value<0.0001), **medical questions** (FC 1.4,1 p-value<0.0001), **emotional statements** (FC 1.87, p-value<0.0001), and **partnership facilitation statements** (FC 3.12 p-value<0.0001), controlling for visit language, provider and patient gender, patient age, visit length, patient education, faculty status, comorbidity count, provider seen at visit was patient’s PCP, and whether a patient was accompanied at clinic visit by a caregiver (yes/no), as well as random effects for providers and clinic visits. Factor change coefficients represent the ratio of clinician expressed to corresponding interpreter-as-clinician conveyed statements. - No association between provider statements and interpreter-as-provider statements about **psychosocial/lifestyle information** or **psychosocial/lifestyle questions**. - Significant association between a higher amount of patient statements (compared to interpreter-as-patient statements) about **medical information** (FC 1.39, p-value<0.001), **psychosocial/lifestyle information** (FC 1.51, p-value<0.05), **emotional statements** (FC 1.53, p-value <0.0001), and **partnership facilitation** (FC 1.60, p-value<0.001), controlling for visit language, provider and patient gender, patient age, visit length, patient education, faculty status, comorbidity count, provider seen at visit was patient’s PCP, and whether a patient was accompanied at clinic visit by a caregiver (yes/no), as well as random effects for providers and clinic visits. Factor change coefficients represent the ratio of patient expressed to corresponding interpreter-as-patient conveyed statements. - Significant association between a lower amount of patient statements (compared to interpreter-as-patient statements) about **medical questions** (FC 0.80, p-value<0.01), controlling for visit language, provider and patient gender, patient age, visit length, patient education, faculty status, comorbidity count, provider seen at visit was patient’s PCP, and whether a patient was accompanied at clinic visit by a caregiver (yes/no), as well as random effects for providers and clinic visits. - No association between patient statements and interpreter-as-patient statements about **psychosocial/lifestyle questions**. - This means that the provider said 2.26 times more statements and the patient made 1.74 times more statements than the interpreter conveyed among all three language preferences (Cantonese, Mandarin, Spanish). - There was also a significant speaker-by-language interaction found, indicating that the factor change coefficient for Spanish-language visits was significantly larger than those of the other two languages: Cantonese-language visits (FC 2.85 Spanish-language visits vs 2.02, p<0.01) and Mandaring-language visits (2.85 Spanish-language visits vs 2.00, p<0.01). This means that Spanish-preferring patients made 2.85 times more statements than the interpreter conveyed. - Significant association for the speaker-by-language interaction (with higher amount of statements for Spanish-preferring patient visits) compared to interpreter-as-provider statements about **medical questions** (Spanish FC 2.05, Cantonese FC 1.19, Mandarin FC 1.16; p-value<0.001) and **emotional talk** (Spanish FC 2.62, Cantonese FC 1.64, Mandarin FC 1.53 p-value<0.001), controlling for visit language, provider and patient gender, patient age, visit length, patient education, faculty status, comorbidity count, provider seen at visit was patient’s PCP, and whether a patient was accompanied at clinic visit by a caregiver (yes/no), as well as random effects for providers and clinic visits. - Significant association between speaker-by-language interaction (with higher amount of statements for Spanish-preferring patients) compared to interpreter-as-patient statements about **emotional statements** (Spanish FC 2.05 p-value <0.001, Cantonese FC 1.28 p-value <0.05, Mandarin FC 1.36 p-value<0.01) and **partnership facilitation** (Spanish FC 2.0 p-value <0.01, Mandarin FC 1.77 p-value <0.001), controlling for visit language, provider and patient gender, patient age, visit length, patient education, faculty status, comorbidity count, provider seen at visit was patient’s PCP, and whether a patient was accompanied at clinic visit by a caregiver (yes/no), as well as random effects for providers and clinic visits. - Significant association in interpreter conveyed statements and worse **patient-centered dialogue** (mean difference 0.12, p-value <0.0001), controlling for visit language, provider and patient gender, patient age, visit length, patient education, faculty status, comorbidity count, provider seen at visit was patient’s PCP, and whether a patient was accompanied at clinic visit by a caregiver (yes/no), as well as random effects for providers and clinic visits. - Patients rated **interpreter listening** between 2 (or fair) and 5 (or excellent), with an average adjusted score of 3.8 (SD 0.9), controlling for visit language, provider and patient gender, patient age, visit length, patient education, faculty status, comorbidity count, provider seen at visit was patient’s PCP, and whether a patient was accompanied at clinic visit by a caregiver (yes/no), as well as random effects for providers and clinic visits. |
| Welty 2012 | **Associations between *Spanish language preference* and worse patient experience, i.e., higher likelihood of problems getting an appointment, and lower likelihood of being comfortable asking nurse questions, understanding nurses, and having medical problems resolved by end of the visit; no association between *Spanish language preference* and communication with the doctor, friendliness of staff and liking care received.**   - Significant associations between Spanish-preference and higher likelihood of reporting **problems getting an appointment** (OR=2.06, CI 1.4-3.1, marginal effect +18%), controlling for reason for visit, clinic location, and time spent in the waiting room. - Significant associations between Spanish-preference and lower likelihood of reporting **being comfortable asking questions to the nurse** (OR=0.21, CI 0.1-0.78, marginal effect -5.2%), **understanding health information/instructions from the nurses** (OR=0.22, CI 0.1-0.9, marginal effect -0.9%), and that **medical problems were fully taken care of at the end of their visit (i.e. resolved)** (OR=0.32, CI 0.2-0.5, marginal effect -15%), controlling for reason for visit, clinic location, and time spent in the waiting room. - No difference found between Spanish versus English preference and **being comfortable asking questions of the doctor, understanding health information/instructions from doctors, and friendliness of front desk staff, friendliness of medical staff,** and **liking care received at clinic**. - Spanish-preferring patients had double the average wait time (as compared to English-preferring patients) (unadjusted mean: 52.2 minutes versus 25.6 min; p-value< .001); which may be due to waiting time for interpreters |
|  | **Pediatric Primary Care** |
| Arauz Boudreau 2010 | **Associations between *provider’s self-reported cultural competence* and better pediatric care experiences (i.e., more family centered care, more helpfulness of care), whereas no association between *language concordance* and pediatric care experiences (i.e., provider communication, family-centered care, helpfulness of care).**   - No significant association between *language concordance* and pediatric care experience (i.e., provider communication (including anticipatory guidance and parental education topics discussed; anticipatory guidance and parental education needs met; assessment of family risk factors), family-centered care, and helpfulness of care provided), controlling for respondent’s education, marital status, child’s insurance status, respondent’s years in the United States, and site of care. - Positive, significant associations between *providers self-reported cultural competency* (i.e., those who self-rated extremely or very effective in caring for Latino patients/families) and better patience experiences (i.e., more **family centered care** (+9.9 points, p-value 0.02, all scales 0-100) and more **helpfulness of care provided** (+16.3 points, p-value 0.02)). - No significant association between providers self-reported cultural competency and provider communication. |
| Clemans-Cope 2007 | **Associations between *being foreign-born parents (who are either English- or Spanish-preferring)* and worse pediatric care experience (i.e., more provider communication problems with child’s care), whereas no association between *Spanish-preferring US-born parents* (as compared to English-preferring US born parents) and likelihood of reporting provider communication problems.**   - No association between *Spanish-preferring US-born parents* (i.e., US born parent who responded to the survey in Spanish) (as compared to US born parents who responded to the survey in English) and likelihood of reporting provider communication problems (i.e., based on CAHPS items of provider never/sometimes (compared to usually/always) listen carefully and explain things) (0.043, p-value=>0.10), controlling for child’s race and ethnicity, and dummy variables for four categories of child’s age, three categories of family structure, three or more children in household, thirteen oversampled states, and survey year. - Significant association between *foreign-born Spanish-preferring parents* (i.e., being foreign born parent who responded to the survey in Spanish) and *foreign-born English-preferring parents* (i.e., being a foreign-born parent who responded to the survey in Spanish) (as compared to US-born parents with English language preference) and likelihood of reporting provider communication problems (i.e., based on CAHPS items of provider never/sometimes (compared to usually/always) listen carefully and explain things) (0.118, p-value=<0.001 and 0.064, p-value=<0.001, respectively), controlling for child’s race and ethnicity, and dummy variables for four categories of child’s age, three categories of family structure, three or more children in household, thirteen oversampled states, and survey year. |
| Flower 2017 | **Associations between *Spanish language preference* and worse patient experience (i.e., worse provider communication about child’s care), whereas no association between *Spanish language preference* and staff communication. Also, no association between *use of* *interpreter* and provider communication about child’s care.**   - Significant association between *Spanish language preference* (as compared to English language preference for Latinos and Non-Latinos) and worse **provider communication i.e., greeted in a way that made me feel comfortable** [English-preferring Latino Incidence Rate Ratio (IRR)=1.29 95% CI (1.13, 1.48); English-preferring Non-Latino IRR=1.15 95% CI (1.02, 1.29)], **treated with respect** [English-preferring Latino IRR=1.24 95%CI (1.11, 1.40); English-preferring Non-Latino IRR=1.14 95% CI (1.03, 1.27)], **interested in ideas about my health** [English-preferring Latino IRR=1.27 95%CI (1.11, 1.45); English-preferring Non-Latino IRR=1.16 95% CI (1.04, 1.31)], **gave as much information as wanted** [English-preferring Latino IRR=1.18 95%CI (1.05, 1.32); English-preferring Non-Latino IRR=1.11 95% CI (1.01, 1.23)], **discussed next steps, including any follow-up plans** [English-preferring Latino IRR=1.20 95%CI (1.06, 1.37); English-preferring Non-Latino IRR=1.14 95% CI (1.03, 1.28)], **spent right amount of time** [English-preferring Latino IRR=1.24 95%CI (1.08, 1.42); English-preferring Non-Latino IRR=1.13 95% CI (1.01, 1.27)], controlling for primary parent age, parent education, household income, and site. - No association between *Spanish language preference* and **staff communication**, **(i.e.,** **treated with respect)** [English-preferring Latino IRR=1.22 95%CI (1.08, 1.38); English-preferring Non-Latino IRR=1.09 95% CI (0.98, 1.21)], controlling for primary parent age, parent education, household income, and site. - No association between *use of an interpreter* and **provider communication about child’s care**, controlling for primary parent age, parent education, household income, site of enrollment. |
| Krugman 2009 | Differences in **pediatric care experiences** **by *Spanish language preference* found better provider communication for Spanish-preferring parents (as compared to English-preferring parents) and no differences for access, longitudinal continuity, contextual knowledge, comprehensive care, and coordination.**   - Positive, significant difference between *Spanish-preferring* parents (and English-preferring parents) and **provider communication** [Mean Score=92.7, 95%CI (89.0, 96.3), p-value<0.001], controlling for race, parental education, age of child, number of visits to site, ability to identify provider, and random site effects. - No significant associations between *Spanish-preferring* parents (and English-preferring parents) and **access, longitudinal continuity, contextual knowledge, comprehensive care, and coordination**. |
| Morales 2006 | **Associations between *use of interpreter (i.e., needing interpreter and always having one)* and better parent experience of child’s care (i.e., provider and staff communication and access to care).**   - Significant associations between *needed an interpreter and always had one* and better **provider and office staff communication** (3.80 p-value <0.01) and **access to care** (1.80, p-value<0.01) for Hispanic parents, controlling for parent age, parent education, survey language, child age, and child health status, health plan, race/ethnicity, and survey year and clustering within health plans. |
|  | **Primary Care for Adult Diabetes Patients** |
| Detz 2014 | **Associations between *language concordance* and better patient experience (i.e., interpersonal processes of care).**   - Significant associations between *language concordance* and better interpersonal processes of care (IPC) (i.e., **clarity of communication** (Concordant vs Discordant: 51% vs 27%, p-value <0.05), **elicited concerns and responded** (61%, vs 38%, p-value <0.05), **explained results** (69% vs 41%, p-value <0.05), **respectful and compassionate** (59% vs 35%, p-value <0.05), **decision making and working together** (35% vs 20%, p-value <0.05), controlling for age, gender, education, and income. |
| Rodriguez 2010 | No differences between ***language concordance* and patient experiences of diabetic patients** **overall or** **by usual care sites (community health centers versus private primary care practices).**   - No association between *language concordance* and the patient experience of diabetic patients (i.e., problematic care and overall rating of care) overall and by usual care sites (community health centers versus private primary care practices), controlling for controlling for age, gender, acculturation, spirituality, income, education, self-rated physical health, depression, insurance/utilization status, use of folk healer, language discordance and region. |
| Sudore 2009 | **Associations between *language concordance* and better patient experience (i.e., provider communication) and associations between *limited health literacy* and worse patient experience (i.e., provider communication) for Spanish-concordant patients.**   - Significant association between Spanish-discordant participants (as compared to English-preferring patients) and **poor proactive communication** (OR 2.44; 95% CI, 1.55-3.84) and both Spanish-concordant (OR 1.7; 95% CI, 1.12-2.59) and discordant (OR 3.60; 95% CI, 2.27-5.70) participants (as compared to English-preferring patients) and **poor interactive communication**, adjusting for age, race, gender, education, site, and language concordance, clustered by physician; health literacy was excluded. - Significant association between both Spanish-concordant (OR 1.59; 95% CI, 1.04-2.44) and Spanish-discordant (OR 3.37; 95% CI, 2.12-5.37) participants (as compared to English-preferring patients) and **poor interactive communication** as well as Spanish-discordant participants (as compared to English-preferring patients) and **poor proactive communication** (OR 2.21; 95% CI 1.39-3.52), adjusting for age, race, gender, education, site, language concordance and health literacy clustered by physician. - Significant association between **limited health literacy** (compared to adequate health literacy) and **poor receptive communication** (OR 1.95; 95% CI 1.31-2.90) and **poor proactive communication** (OR 1.82; 95% CI 1.22-2.72), adjusting for age, race, gender, education, site, language concordance, and health literacy clustered by physician. - Significant association in stratified analyses between **limited health literacy** and **poor proactive communication** and **poor interactive communication** (p-value <0.05) for Spanish-concordant patients. - Null association in stratified models between **limited health literacy** and **poor proactive communication** and **poor interactive communication** (p-value <0.05) for Spanish-discordant patients. |
| Zamudio 2017 | **Descriptive themes about the relationship between *language concordance* and patient experience for Latinos were mixed.**   - Most Latino patients with Spanish-speaking providers re­ported advantages of language con­cordance, indicating shared language facilitated communication and the increased comfort resulted in full disclosure of health informa­tion. - Language concordance was not seen as absolutely necessary among the patients with English-speaking providers. Trust in the patient-provider relationship with discordant language led many Latino patients to remain with English-speaking providers who treated them well. - Latino patients with either language concordant or discor­dant providers reported reliance on family or other intermediaries to close communica­tion gaps. - Regardless of language concordance, deference to physician expertise and authority led to visit expectations for Latino patients, specifically that it is the doctor’s job to know what to ask and that visits were intended to address specific, often symptom-driven problems. |
|  | **Primary Care Focused on Mental Health** |
| August 2011 | **No association between *language concordance* and better patient experience (i.e., patient-provider discussion of mental health needs) for Latinos.**   - No association between the interaction of race/ethnicity and *Spanish-language concordance* for Latinos and **patient-provider discussions of mental health needs**, adjusting for age, sex, marital status, education, insurance status, nativity, and psychological distress. - No association between *Spanish-language concordance* or *language discordance* and **patient-provider discussion of mental health needs** for Latinos, adjusting for age, sex, marital status, education, insurance status nativity, and psychological distress. |
|  | **Primary Care interfacing with Specialty Care** |
| Song 2022 | **No association between *interpreter use* and better patient experience (i.e., specialist/ophthalmologist referral).**   - No association between *needing an interpreter* and the FQHC provider making an ophthalmology referral following teleretinal imaging (TRI) (OR 1.05; 95% CI .96-1.15; p-value 0.28), adjusting for ethnicity, need for interpreter services, diabetic nephropathy, diabetic neuropathy, and glomerular filtration rate (GFR). - No association between *needing an interpreter* and ophthalmology visit attendance and between *needing an interpreter* and adherence to recommended ophthalmology exam, regardless of follow-up recommendation based on TRI, adjusting for age, and history of diabetic nephropathy, diabetic neuropathy, hypertension, or proteinuria. |
